# Cryptotanshinone Induces Cell Cycle Arrest and Apoptosis of NSCLC Cells through the PI3K/Akt/GSK-3β Pathway

**DOI:** 10.3390/ijms19092739

**Published:** 2018-09-13

**Authors:** Sang-A Kim, Ok-Hwa Kang, Dong-Yeul Kwon

**Affiliations:** Department of Oriental Pharmacy, College of Pharmacy and Wonkwang Oriental Medicines Research Institute, Wonkwang University, Iksan, Jeonbuk 570-749, Korea; tkddk1532@naver.com

**Keywords:** cryptotanshinone, NSCLC, cell cycle arrest, apoptosis, PI3K/Akt/GSK3β

## Abstract

Cryptotanshinone (CTT) is a natural product and a quinoid diterpene isolated from the root of the Asian medicinal plant, *Salvia miltiorrhizabunge*. Notably, CTT has a variety of anti-cancer actions, including the activation of apoptosis, anti-proliferation, and reduction in angiogenesis. We further investigated the anti-cancer effects of CTT using MTS, LDH, and Annexin V assay, DAPI staining, cell cycle arrest, and Western blot analysis in NSCLC cell lines. NSCLC cells treated with CTT reduced cell growth through PI3K/Akt/GSK3β pathway inhibition, G0/G1 cell cycle arrest, and the activation of apoptosis. CTT induced an increase of caspase-3, caspase-9, poly-ADP-ribose polymerase (PARP), and Bax, as well as inhibition of Bcl-2, survivin, and cellular-inhibitor of apoptosis protein 1 and 2 (cIAP-1 and -2). It also induced G0/G1 phase cell cycle arrest by decreasing the expression of the cyclin A, cyclin D, cyclin E, Cdk 2, and Cdk 4. These results highlight anti-proliferation the latent of CTT as natural therapeutic agent for NSCLC. Therefore, we investigated the possibility of CTT as an anti-cancer agent by comparing with GF, which is a representative anti-cancer drug.

## 1. Introduction

Lung cancer has two major subtypes: non-small-cell lung cancer (NSCLC) and small-cell lung cancer (SCLC) [1]. Eighty to 85% of lung cancer is constituted as NSCLC but only 5% of patients with NSCLC survive after the fourth stage. NSCLC therapies, such as surgery, chemotherapy, irradiation, etc., have been used [2,3]; however, only 15–30% of lung cancer patients are cured with chemotherapy and the symptoms of cancer can even return shortly after surgery due to the metastatic nature of the disease [4,5]. In this case, natural products are attracting attention as anti-cancer drugs because of fewer side effects and higher efficacies than conventional chemotherapy anticancer drugs [6].

Cryptotanshinone (CTT) is a natural product isolated from the root of the Asian medicinal plant, *Salvia miltiorrhizabunge*. Published studies have reported that CTT has a various pharmacological effects, such as anti-inflammation [7], anti-diabetes [8], and anti-cancer effects of CTT [9,10,11,12]. Recently, a paper has revealed that CTT induced apoptosis in NSCLC through PTEN-mediated inhibition of the PI3K/Akt pathway [9]; however, no studies have been investigated on the anti-cancer effects of CTT through the PI3K/Akt/ GSK-3β pathway and the comparison with existing anti-cancer drugs.

Gefitinib which is epidermal growth factor receptor tyrosine kinase inhibitors (EGFR-TKIs) was selected as a comparative group for the anti-cancer efficacy comparison of CTT. These days, representative chemotherapeutic agents used in NSCLC are epidermal growth factor receptor tyrosine kinase inhibitors (EGFR-TKIs) [13]. EGFR-TKIs therapies show considerable promise, but people who use docetaxel or cisplatin to treat EGFR antigens exhibit a drug resistance to EGFR-TKIs, and they also have the significant side effect of excessive cytotoxicity. These negative side effects are clear in all EGFR-TKIs, but one of them, Gefitinib (GF), prolongs progression-free survival with acceptable toxicity, compared to other standard chemotherapy agents [14,15]. Therefore, we selected GF as a comparative group.

Taken together, we considered that the apoptosis effect of CTT on non-small cell lung cancer is related to the pathway of PI3K/AKT/GSK3B, and that the apoptosis effect of the previously known CTT needs to be compared with conventional chemotherapy. Thus, our present study aimed to further investigate the pathways involved in the NSCLC of CTT and to demonstrate its efficacy by comparing it with existing anti-cancer drug.

## 2. Results

### 2.1. CTT Inhibited the Viability of A549 and H460 Cells

**Figure 1:**
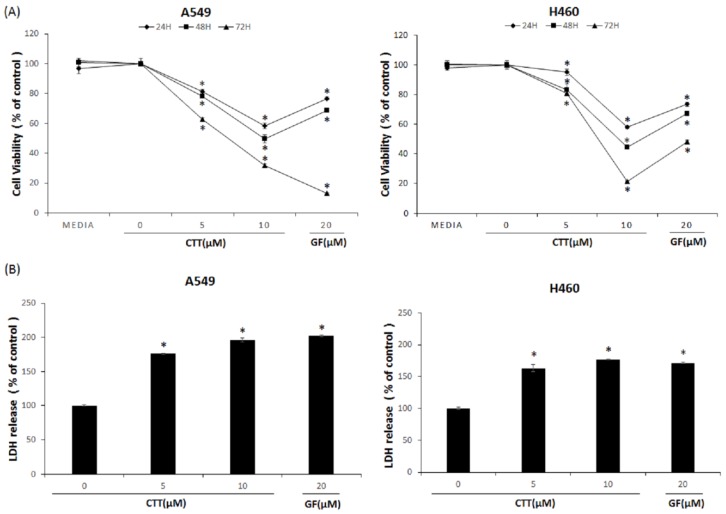
Effects of CTT treatment on cytotoxicity in A549 and H460 cells. (**A**) Cell viability was evaluated through MTS assay after 24, 48, and 72 h treatment with 0, 5, or 10 μM of CTT or 20 μM GF (clinical anticancer drug). * *p* < 0.05 compared to the control group. (**B**) LDH release was assessed using an LDH assay kit after 24 h treatment with 0, 5, or 10 μM of CTT or 20 μM GF (clinical anticancer drug). * *p* < 0.05 compared to the 0 μM of CTT group. The data represent each of the three independent experiments. Significant differences for the treated groups were determined by Duncan’s test for multiple comparisons. Values are represented as the mean ± SD from each experiment.

MTS assays were performed to evaluate the effects of CTT on the viability of A549 and H460 cells. As shown in Figure 1A, CTT markedly decreased the viability of A549 and H460 cells in a dose- and time-dependent manner. Notably, the cytotoxic effects of 10 μM CTT on NSCLC cells were higher than GF at both 24 h and 48 h. Furthermore, LDH release in the culture supernatant was increased in a dose-dependent manner after treatment of CTT for 24 h (Figure 1B). Thus, these results indicate that CTT inhibited the viability of A549 and H460 cells.

### 2.2. CTT Increased A549 and H460 Apoptosis

**Figure 2:**
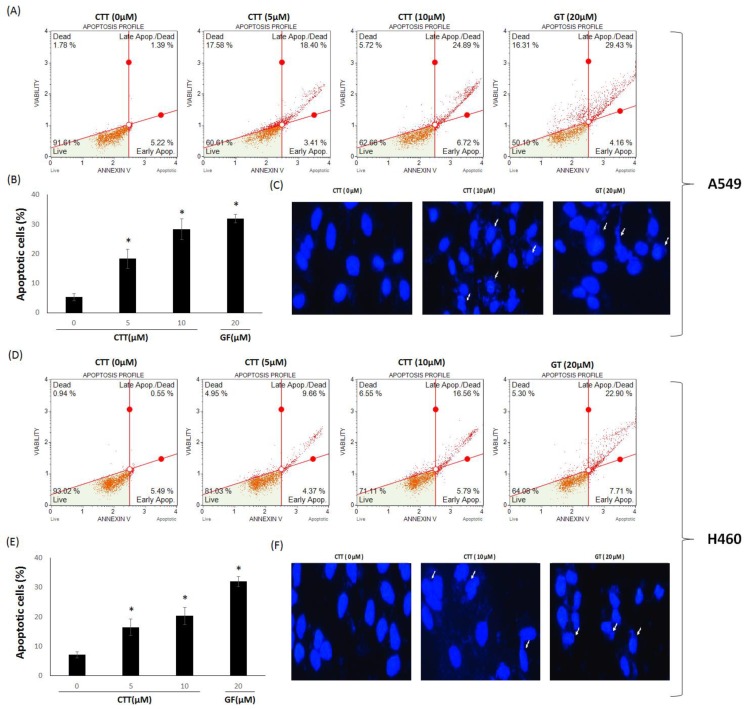
Effects of CTT treatment on apoptosis in A549 and H460 cells. (**A**,**D**) The cells were treated with 0, 5, or 10 μM of CTT or 20 μM GF (clinical anticancer drug) and stained with Annexin V, PI. After staining, flow cytometry was performed to determine apoptosis. (**B**,**E**) The histograms of the apoptotic cells were analyzed with a MUSE™ cell analyzer. (**C**,**F**) Nuclear condensation and fragmentation after 24 h of treatment with 10 μM CTT or 20 μM GF (clinical anticancer drug), stained with DAPI and visualized by fluorescent microscope (magnification, 400×). * *p* < 0.05 compared to the 0 μM of CTT group. The data and images each represent one of the three independent experiments. Significant differences for the treated groups were determined by Duncan’s test for multiple comparisons. Values represented as mean ± SD from each experiment.

To confirm whether the effects of CTT on the cytotoxicity of NSCLC cells were related to apoptosis, an Annexin V assay was performed. As shown in Figure 2A,B,D,E, CTT dose-dependently increased apoptosis in A549 and H460, but did not increase apoptosis more than GF. The cells were stained with DAPI to better represent the obvious morphological changes related to apoptosis (Figure 2C,F). The white arrow markers show nuclear condensation and fragmentation. Thus, these results indicate that CTT induced cytotoxicity by apoptosis.

### 2.3. CTT Affected the Expression Levels of Apoptosis-Related Proteins in A549 and H460 Cells

**Figure 3:**
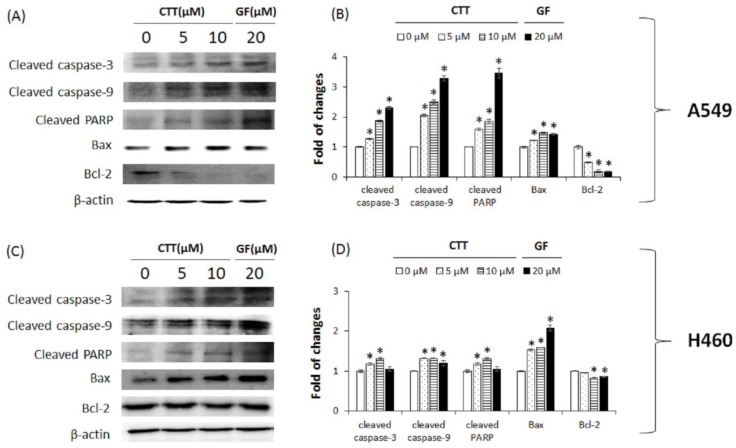
Effects of CTT treatment on the expression of apoptosis-related pathway proteins in A549 and H460 cells. (**A**,**C**) After treatment with 0, 5, or 10 μM of CTT or 20 μM GF (clinical anticancer drug) for 20h, the protein levels of cleaved caspase-3, cleaved caspase-9, cleaved PARP, Bax, and Bcl-2 were determined through western blotting. (**B**,**D**) The calculations of the results were normalized against β-actin. * *p* < 0.05 compared to the 0 μM of CTT group. The data and images represent each of the three independent experiments. Significant differences for the treated groups were determined by Duncan’s test for multiple comparisons. Values are represented as the mean ± SD from each experiment.

To elucidate the mechanism of CTT-mediated apoptosis, apoptosis-related protein expression was measured through western blot analysis. After treatment of CTT in NSCLC cells, the levels of cleaved caspase-3, cleaved caspase-9, cleaved PARP, and Bax were increased. Conversely, the levels of Bcl-2, anti-apoptotic protein, were decreased (Figure 3A–D). A549 cells showed more appropriate increase or decrease results than H460 cells in apoptosis-related protein. These results indicate that CTT-induced apoptosis is associated with activating the apoptosis pathway and inhibiting Bcl-2.

### 2.4. CTT Induced G0/G1 Cell Cycle Arrest in A549 and H460 Cells

**Figure 4:**
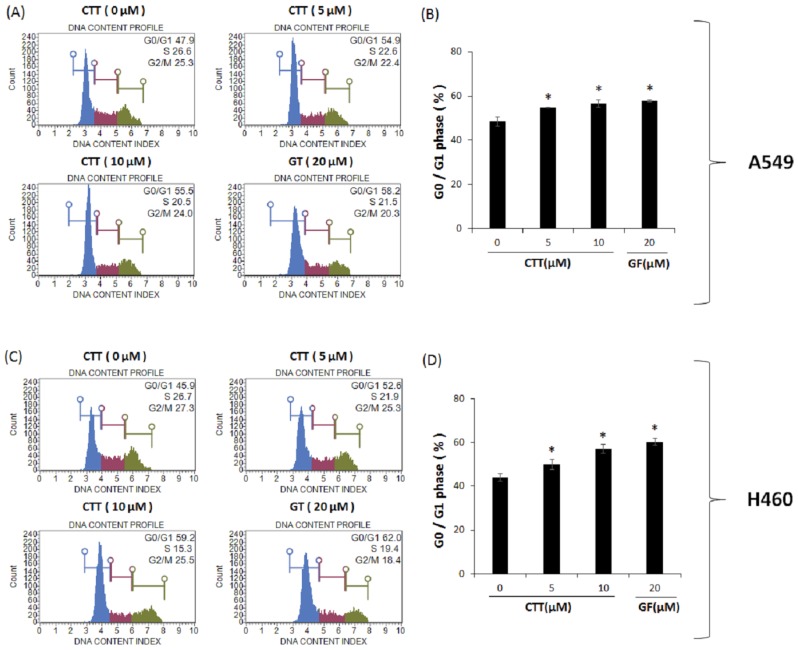
Effects of CTT treatment on G0/G1 phase arrest in A549 and H460 cells. (**A**,**C**) The cell cycle distribution after 16h treatment with 0, 5, or 10 μM of CTT or 20 μM GF (clinical anticancer drug) was measured using flow cytometry. (**B**,**D**) The histogram of the rate of G0/G1 phase cell was analyzed with a MUSE™ cell analyzer. * *p* < 0.05 compared to the 0 μM of CTT group. The data represent each of the three independent experiments. Significant differences for the treated groups were determined by Duncan’s test for multiple comparisons. Values are represented as the mean ± SD from each experiment.

To investigate whether the increased apoptosis is related to cell cycle arrest, the number of cells in the G0/G1 phases were analyzed through flow cytometry. The results in CTT-treated A549 (Figure 4A,B) and H460 (Figure 4C,D) showed that the percentage of cells in the G0/G1 phases increased significantly next to non-treated cells, but did not increase cell cycle arrest more than GF. These results demonstrate that apoptosis induced by CTT is related to cell cycle arrest.

### 2.5. CTT Affected the Expression Levels of Proteins Related to Cell Cycle Regulatory in A549 and H460 Cells

**Figure 5:**
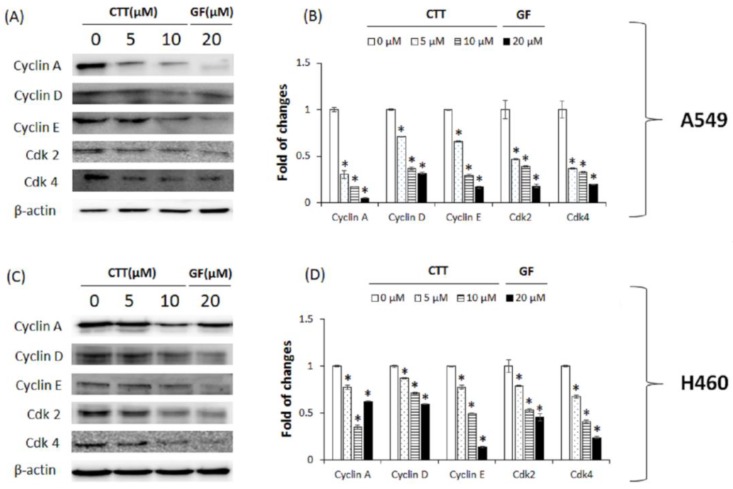
Effects of CTT treatment on the expression of G1 and S cell cycle checkpoint proteins in A549 and H460 cells. (**A**,**C**) After treatment with 0, 5, or 10 μM of CTT or 20 μM GF (clinical anticancer drug) for 20 h, the protein levels of cyclin A, D, and E and Cdk 2 and 4 were determined through Western blotting. (**B**,**D**) The calculations of the results were normalized against β-actin. * *p* < 0.05 compared to the 0 μM of CTT group. The data and images represent each of the three independent experiments. Significant differences for the treated groups were determined by Duncan’s test for multiple comparisons. Values are represented as the mean ± SD from each experiment.

To verify the mechanism of CTT on G0/G1 arrest, we analyzed the expression levels of proteins involved in the G1 and S phase regulators through Western blot analysis. As shown in Figure 5A,B (A549) and Figure 5C,D (H460), the CTT-treated group showed not only a significant decrease in the expression of the G1 phase (cyclin D, E, and Cdk 4), but also a significant decrease in the expression of the S phase (cyclin A, Cdk 2) compared to the non-treated group. These results reveal that the effects of CTT on G0/G1 phase arrest were induced via changing the expression of proteins related to cell cycle regulation.

### 2.6. CTT-Modulated IAP Family in A549 and H460 Cells

**Figure 6:**
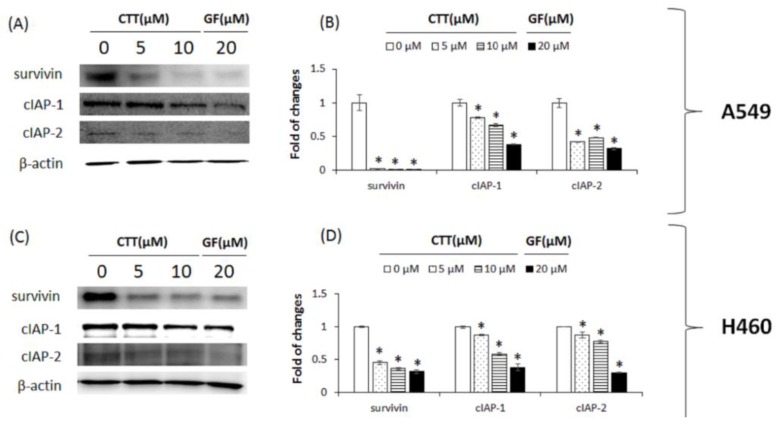
Effects of CTT treatment on the expression of IAP family proteins in A549 and H460 cells. (**A**,**C**) After treatment with 0, 5, or 10 μM of CTT or 20 μM GF (clinical anticancer drug) for 20 h, the protein levels of cIAP-1, cIAP-2, and survivin were determined through western blotting. (**B**,**D**) The calculations of the results were normalized against β-actin. * *p* < 0.05 compared to the 0 μM of CTT group. The data and images represent each of the three independent experiments. Significant differences for the treated groups were determined by Duncan’s test for multiple comparisons. Values are represented as the mean ± SD from each experiment.

Western blot analysis was performed in order to further elucidate the members of the IAP family, which play an important role in anti-apoptosis in NSCLC cells. After CTT treatment, protein expression levels of the IAP family, such as those of survivin, cIAP-1, and cIAP-2, were significantly decreased (Figure 6A–D). These results further indicate that the apoptotic effects of CTT in NSCLC were activated by inhibiting not only the Bcl-2 family, but also the IAP family.

### 2.7. CTT Inhibited the PI3K/Akt/GSK-3β Pathway

**Figure 7:**
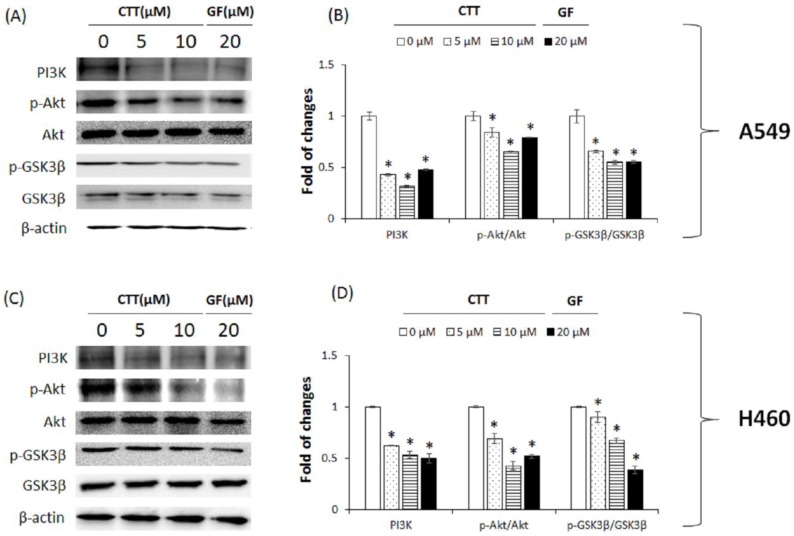
Effects of CTT treatment on the expression of PI3K/Akt/GSK-3β pathway proteins in A549 and H460 cells. (**A**,**C**) After treatment with 0, 5, or 10 μM of CTT or 20 μM GF (clinical anticancer drug) for 20 h, the protein levels of PI3K, p-Akt/Akt, p-GSK3β/GSK3β were determined through western blotting. (**B**,**D**) The calculations of the results were normalized against specific antibodies or β-actin. * *p* < 0.05 compared to the 0 μM of CTT group. Significant differences for the treated groups were determined by Duncan’s test for multiple comparisons. Values are represented as the mean ± SD from each experiment.

We performed western blot analysis to examine the PI3K/Akt/GSK3β pathway by measuring the protein expression levels of PI3K, p-Akt/Akt, and p-GSK3β/GSK3β. After CTT treatment, we observed that CTT significantly diminished the expression of PI3K, p-Akt/Akt, p-GSK3β/GSK3β in both cell lines in comparison to the non-treated cells (Figure 7A–D). These results showed that CTT inhibited the protein expression levels of the PI3K/Akt/GSK-3β pathway.

## 3. Discussion

Cryptotanshinone (CTT) is a natural product and a quinoid diterpene isolated from the root of the Asian medicinal plant, *Salvia miltiorrhizabunge*. Although previous studies have demonstrated the effect of the active ingredient such as tanshinone I extracted from *Salvia miltiorrhizabunge* in NSCLC [16,17]; but studies on cryptotanshinone are not enough. Therefore, we further investigated the mechanism of CTT on representative NSCLC cells, A549 and H460 cell lines. As can be seen from the results, CTT significantly increased cytotoxicity through induction of apoptosis and cell cycle arrest, which was conducted to be the result of PI3K/AKT/GSK3B pathway regulation.

Cell cycle arrest is one way to eliminate cancer cells [18]. In brief, the cell cycle progression is a key process for replicating cells that is accomplished through a series of checkpoints. These checkpoints are activated by DNA damage. In this case, the growth arrest caused by checkpoints allows the cell to repair the damage. If the damage cannot be repaired, the cell is eliminated through apoptosis [19].

Apoptosis is important for maintaining tissue homeostasis. The proper regulation of apoptosis elimination inhibits the general growth of cancer cells [20]. Generally, apoptosis occurs through the extrinsic pathway or intrinsic pathway. These pathways were converged by executioner caspases at the final pathway of apoptosis [21]. Many natural products have been found to induce apoptosis through the intrinsic pathway [22]. The intrinsic pathway is mediated by mitochondria [23]. When DNA damage or oncogene activation occurred, the intrinsic pathway is triggered [24] and the overall intrinsic pathway is regulated by the Bcl-2 family of proteins [25]. The Bcl-2 family proteins which are anti-apoptotic member, Bcl-2, and pro-apoptotic member, Bax are known as key regulators of apoptosis [26].

Cytochrome c, the release of mitochondrial proteins, is mediated by Bax [27]. An increased Bax and decreased Bcl-2 cause a release of cytochrome c from mitochondria to the cytosol where it activates caspase-9 [28] which subsequently activates the executioner caspase-3 [29]. The executioner caspases quickly begin to cleave such as PARP leading to apoptosis [30]. Inhibitor of apoptosis (IAP) proteins regulate negatively caspases and cell death [31]. IAPs such as survivin, cIAP1 and cIAP-2, are a group of structurally-related proteins that block apoptosis either by binding and inhibiting caspases [32].

Previously-published studies have indicated that the PI3K/Akt cell signaling pathway suppresses apoptosis and implements cell proliferation, cycle progression, and metastasis of cancer [33,34,35,36,37]. GSK3β, which is an Akt substrate, is negatively regulated by Akt activity. GSK3β has two capabilities according to whether it is activated or phosphorylated. Activated GSK3β is involved in pro-apoptosis but phosphorylated GSK3β, because of its Akt activity, interrupt the apoptotic activity [38,39].

Results from Figure 1 showed that CTT markedly decreased the viability of A549 and H460 cells in a dose- and time-dependent manner. As shown in Figure 2, Figure 3 and Figure 6, CTT significantly increased apoptosis in a dose-dependent manner in A549 and H460, indicating that anti-apoptotic proteins such as survivin, cIAP-1, cAP-2, and Bcl-2 are reduced at the protein level whereas cleaved caspase-3, cleaved caspase-9, cleaved PARP, and Bax. These data indicate that CTT induced cytotoxicity through apoptosis, which is triggered through intrinsic pathway mediated by mitochondria. In addition, our results showed that CTT induced G0/G1 cell cycle arrest in A549 and H460 cells (Figure 4) and diminished the protein level of each checkpoints which are cyclin D/Cdk 4 complex (from G0 into G1 phase), cyclin E/Cdk 2 complex (from G1 to S phase transition), and cyclin A (binding with Cdk 2 during S phase) (Figure 5) [19]. These results indicated that the apoptosis effect of CTT is related to cell cycle arrest.

**Figure 8:**
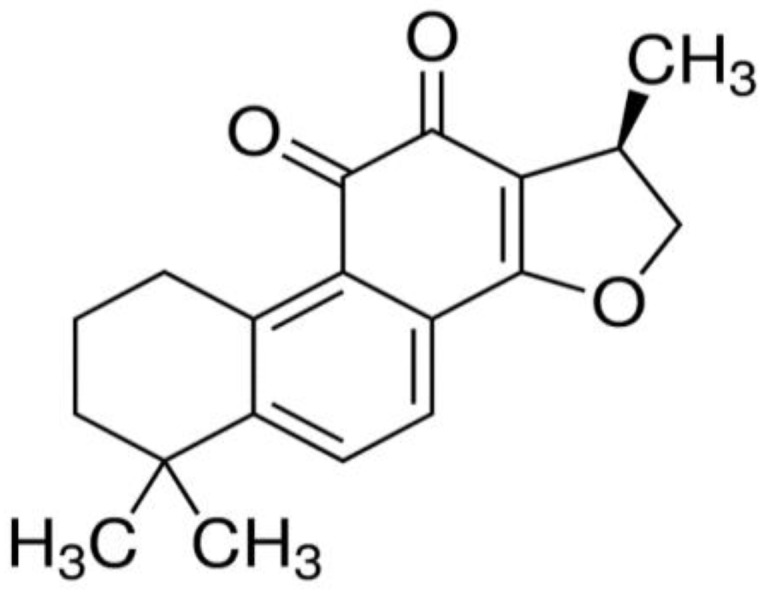
Structure of cryptotanshinone (CTT).

After that, we investigated at the mechanism of CTT. As presented in Figure 8, our results showed markedly diminished protein expression in PI3K, p-Akt/Akt, and p-GSK-3β/GSK-3β signaling pathway factors. These results mean that CTT induces apoptosis by inhibiting phosphorylation of Akt and sequentially inhibiting phosphorylation of gsk3b.

Altogether, this study certifies the anticancer effect of CTT in NSCLC by inducing G0/G1 cell cycle arrest and apoptosis through PI3K/Akt/GSK3β signaling pathway inhibition. Thus, CTT could be a potential therapeutic natural agent for targeting the PI3K/Akt/GSK3β in NSCLC.

## 4. Materials and Methods

### 4.1. Reagents

Fetal bovine serum (FBS), penicillin/streptomycin were purchased from Hyclone (Logan, UT, USA). 3-(4,5-dimethylthiazol-2-yl)-5-(3-carboxymethoxyphenyl)-2-(4-sulfophenyl)-2H-tetrazolium (MTS), bovine serum albumin (BSA), dimethyl sulfoxide (DMSO), 4′,6-diamidino-2-phenylindole (DAPI), formaldehyde, Cryptotanshinone (CTT), and Gefitinib (GF) were purchased from Sigma-Aldrich (St. Louis, MO, USA). A lysis buffer was purchased from iNtRon Biotech (Seoul, Korea). Antibodies for cleaved caspase-3 (#9661), cleaved caspase-9 (#9505), cleaved PARP (#9541), survivin (#2808), Cdk4 (#12790), Cyclin D1 (#2978), Phospho-Akt (#4058), and Akt (#9272) were purchased from Cell Signaling (Cell Signaling Technology, Beverly, MA, USA). Cdk2 (sc-6248), Cyclin A (sc-751), Cyclin E (sc-198), Bcl-2 (sc-7382), Bax (sc-7480), PI3K (sc-602), Phospho-GSK-3β (sc-81494), GSK-3β (sc-81462), cIAP-1 (sc-271419), cIAP-2 (sc-517317), β-actin (sc-47778), and peroxidase-conjugated secondary rabbit (sc-2357) and mouse (sc-516102) antibodies were purchased from Santa Cruz Biotechnology, Inc. (Santa Cruz, CA, USA).

### 4.2. Preparation of CTT and GF

Cryptotanshinone (CTT) (C5624, ≥98%(HPLC)) (Figure 8) was purchased from Sigma-Aldrich. CTT was dissolved in dimethyl sulfoxide (DMSO) to a stock concentration of 100 mM and stored at 4 °C. The final concentration of DMSO in all experiments did not exceed 0.1% and the final CTT solutions were all diluted in the fresh culture medium. Additionally, the concentration of 20 μM GF was dissolved with DMSO and stored at 4 °C.

### 4.3. Cell Culture

The human NSCLC cell line A549 and H460 were obtained from the Korean Cell Line Bank (Seoul, Korea) and grown in RPMI 1640 (Thermo Fischer Scientific, Waltham, MA, USA) containing 10% FBS, 100 units/mL penicillin, and 100 μg/mL streptomycin at 37 °C with 5% CO_2_ in air [40]. We only used each cell lines up to passage 15.

### 4.4. Cell Viability

The effects of CTT on A549 and H460 cell viability were determined through an MTS assay [40]. Cells were seeded in 96-well culture plates (2 × 10^3^ cells/well) and incubated for 24 h. GF was used as a positive control. Following incubation, various concentrations (0, 5, or 10 μM) of CTT or 20μM GF were added and incubated for 24, 48, and 72 h. The medium was replaced with MTS solution and each culture well was optimized with a Micro plate reader (TitertekMultiskan, Flow Laboratories, North Ryde, Australia) at 490 nm. Cell viability was calculated using the Equation (1):

Cell viability (%) = (OD 490 value of CTT treated cells/OD 490 value of untreated cells) × 100(1)

### 4.5. LDH Assay

The effects of CTT on A549 and H460 LDH release were detected using the Pierce^®^ LDH Cytotoxicity Assay Kit (Thermo Fisher Scientific, MA, USA), according to the manufacturer’s instructions. In brief, the cells were seeded in 96-well plates (2 × 10^3^ cells/well) for 24 h, then treated with CTT (0, 5, or 10 μM) or GF (20 μM) for 24 h. Next, the maximum LDH well was added to a 10 μL 10X lysis buffer. After 45 min, the supernatants were transferred into a new 96-well plate, and 50 μL of reaction mixture was added to each well. Finally, the supernatants were incubated for 30 min in room temperature and 50 μL stop solution were put into each well. LDH release were measured at 490 nm and 680 nm of absorbance and calculated using Equation (2):

LDH release (%) = [(OD 490-OD 680 value of CTT treated cells) − (OD 490-OD 680 value of untreated cells)/(OD 490-OD 680 value of maximum LDH cells − OD 490-OD 680 value of untreated cells) × 100](2)

### 4.6. DAPI Staining

Cell nuclear morphology was measured through fluorescence microscopy following DAPI staining [40]. A549 and H460 cells were treated with CTT for 24 h. Cells were then washed with PBS, fixed with 4% formaldehyde and washed 2 times each for 5 min. Next, cells were stained with DAPI, and incubated for 5 min at room temperature away from light. The cells were finally washed with PBS one time and visualized using a fluorescence microscope (Carl Zeiss, Oberkochen, Germany).

### 4.7. Cell Cycle Analysis

Cell cycle components were analyzed with Muse™ cell cycle reagent (EMD Millipore Corp [40]. Billerica, MA, USA). A549 and H460 cells (10 × 10^4^ cells/well) were seeded in six-well plates and treated with CTT (0, 5, or 10 μM) or GF 20 μM for 16 h. After being harvested from the culture medium, cells were washed with 1mL PBS, then fixed with ice-cold fresh 70% EtOH for over 3 h. Next, cells were washed with PBS and had Muse™ cell cycle reagent added to them. Then, cells were incubated at room temperature for 30 min without light. Cells were analyzed with Muse cell analyzer and Muse analysis software (Merck Millipore).

### 4.8. Annexin V Assay

The extent of apoptosis was determined by Muse Annexin V and a dead cell kit (Millipore, Billerica, MA, USA). According to the manufacturer’s procedure, A549 and H460 cells (10 × 10^4^ cells/well) were seeded in 6-well plates and treated with CTT (0, 5, or 10 μM) or GF 20 μM for 24 h. After being harvested from the culture medium, cells were washed with 1 mL PBS and 100 μL of Muse™ Annexin V Dead Cell reagent were added to them. Next, cells were incubated at room temperature for 20 min without light. The apoptosis was then analyzed with Muse cell analyzer and Muse analysis software (EMD Millipore).

### 4.9. Western Blotting

After treatment of A549 and H460 cells with CTT (0, 5, or 10 μM) or GF 20 μM for 20 h, harvested cells were lysed with a lysis buffer (iNtRon Biotech, Seoul, Republic of Korea) for 20 min. The cell lysates were centrifuged for 5 min at 13,000 rpm and protein concentration was determined through bicinchoninic acid (BCA) assay. The cell lysates were mixed with a 3× sample buffer, inserted to SDS gel electrophoresis, and then transferred onto a PVDF membrane (Millipore, Bedford, MA, USA). The membrane was blocked with 5% skim milk or 3% BSA for 1 h and probed overnight at 4 °C with primary antibodies. After a series of washes, the membrane was incubated for 1 h at room temperature with a secondary antibody conjugated with horseradish peroxidase (HRP). Next, the proteins were supplemented with ECL prime Western blotting detection reagents (GE Healthcare, UK). ImageQuant LAS 4000 Mini Biomolecular Imager (GE Healthcare, UK) was used for analyzing the Bands [40].

### 4.10. Statistical Analyses

The statistical analysis was performed using one-way analysis of variance, followed by Duncan’s test for various comparisons. Results are presented as the means ± standard deviations of the independent experiments. All calculations were performed using SPSS Statistics 23 software (SPSS Inc. Chicago, IL, USA). Comparisons with *p* < 0.05 were considered to be statistically significant.
